# Psychopathology without Borders: Transcultural psychiatry and implications in clinical presentation and practice

**DOI:** 10.1192/j.eurpsy.2023.1742

**Published:** 2023-07-19

**Authors:** S. Jesus, A. R. Costa, G. Simões, A. I. Gomes, A. Tarelho, P. Garrido

**Affiliations:** Departamento de Psiquiatria e Saúde Mental, Centro Hospitalar do Baixo Vouga, Aveiro, Portugal

## Abstract

**Introduction:**

Existing as an emerging topic in the field and undergoing constant evolution, Transcultural Psychiatry addresses how social and cultural factors influence mental illness. During the second half of the twentieth century, phenomena such as globalization, massive migrations and immigration, occurring in ever increasing frequency, continue to bring this topic to the forefront of discussion as challenges in the treatment of patients from varying cultural backgrounds emerge. Viewed from the biopsychosocial perspective, culture delineates a framework for the evaluation of various expressions of emotion and behaviour as well as defining the limits of what counts as disorder. As border restrictions are lifted, cases which present with these particularities are bound to increase, necessitating an increased attention to the influence that cultural and social factors play in the psychopathological clinical pictures which may present to the practitioner.

**Objectives:**

The authors aim to briefly explore the concept of transcultural psychiatry and its importance in clinical presentation and practice with recourse to various clinical cases of international patients hospitalized in a Portuguese Psychiatry ward during a two-year period.

**Methods:**

A brief non-systematized literature review was performed based on works most pertinent to the topic discussed. As compliment to the topic, a discussion of various clinical cases of hospitalized international patients is presented.

**Results:**

Culture has been demonstrated to contribute to psychopathological presentations in a variety of forms, solidifying the old adage that ‘no man is an island’ and giving reason to the biopsychosocial approach applied in clinical practice. The impact of sociocultural factors is such that the DSM-5-TR includes in its classification culture-specific syndromes. The cases discussed demonstrate the various nuances necessary not only in exploring psychopathology, but also in implementing appropriate standards of care.

**Conclusions:**

Transcultural psychiatry rises as a relatively recent topic as well as raising important philosophical, theoretical and technical challenges for mental health practitioners. Although existing as a subspecialty, each mental health practitioner should strive to be transcultural, taking into consideration the influence that these factors exert on mental illness. The patient should be evaluated with consideration to their cultural background, as well as not neglecting how the culture of the practitioner may influence the interpretation of psychopathological presentation.

**Disclosure of Interest:**

None Declared

